# *Helicobacter Pylori* stool antigens: post and pre-treatment evalutation of two methods performances in adult’s strains in Algeria

**DOI:** 10.1186/1753-6561-5-S1-P96

**Published:** 2011-01-10

**Authors:** F Kias, F Taleb, F Mouffok, N Matougui, MA Boudjella, Z Guechi, H Berrah, A Bouhadef, KH Bouzid, B Touchene

**Affiliations:** 1 Institut Pasteur d’Algérie, Algiers, Algeria; 2 Laboratoire Algérien de recherche sur Helicobacter, Université d’Alger, Algiers, Algeria

## 

Diagnostic of *H.pylori* infection requires several methods, some of them are currently used in “Institut Pasteur “of Algeria. The aim of this study is to evaluate performances of two enzyme immunoassays used in detection of *H.pylori* using stool Ag before and after eradication, the first one “premier Platinium HpSA, Meridian Diagnostics” use a polyclonal Ac, the other one “IDEA HpStAR, Oxoid” use a monoclonal Ac. The reference test used in this study was UBT which is considered as a gold standard.

More than 1000 stools specimens were studied prospectively between 2000 and 2008, in adult subjects which are more than 16 years old. For each patient two Stools specimens are used, one before eradication and one minimum 04 weeks after treatment. 749 stools specimens were collected before eradication and only 265 ones after.

Our findings show that “IDEA HpStAR, Oxoid” results (specificity: 83%, sensibility: 91% before eradication, 83% after eradication) are better than “Premier Platinium HpSA^®^, Meridian Diagnostics” ones (specificity: 76%, sensibility: 64% before eradication, 56% after eradication). It seems evident that using monoclonal Ac gives more performances to the method, and allows detection of active infection with less of “false positive” also before than after eradication.

